# Geographical Environment Factors and Risk Mapping of Human Cystic Echinococcosis in Western China

**DOI:** 10.3390/ijerph15081729

**Published:** 2018-08-12

**Authors:** Duan Huang, Rendong Li, Juan Qiu, Xiangdong Sun, Ruixia Yuan, Yuanyuan Shi, Yubing Qu, Yingnan Niu

**Affiliations:** 1 Institute of Geodesy and Geophysics, Chinese Academy of Sciences, Wuhan 430077, China; huangduan@asch.whigg.ac.cn (D.H.); lrd@asch.whigg.ac.cn (R.L.); shiyuanyuan13@mails.ucas.ac.cn (Y.S.); quyubing15@mails.ucas.ac.cn (Y.Q.); niuyingnan16@mails.ucas.ac.cn (Y.N.); 2 University of Chinese Academy of Sciences, Beijing 100049, China; 3 China Animal Health and Epidemiology Center, Qingdao 266032, China; 4 Department of Epidemiology and Biostatistics, School of Health Sciences, Wuhan University, Wuhan 420000, China; yuanwhuer@163.com

**Keywords:** human cystic echinococcosis, remote sensing, environment factors, risk mapping, Western China

## Abstract

The study aimed to reveal the risk factors and predict the prevalence of human cystic echinococcosis (CE) in Western China. To do this, we analyzed county-wide data relating to the prevalence of human CE in seven provinces of Western China, along with associated human, natural geographical environmental data. We then used spatial analysis and multiple regression analysis to investigate the correlation between the prevalence of human CE and associated environmental factors and to create a risk map of the disease in the seven provinces. Our analysis showed that grassland area ratio and Tibetan population ratio were independent variables positively correlated with the prevalence of human CE and that gross domestic product (GDP) and land surface temperature (LST; Spring) were negative independent variables. We also created a predictive risk map of human CE that revealed that the high-risk areas were mainly located in the south of Qinghai, the Northwest of Sichuan and most of the Tibet Autonomous Region. Knowledge of the spatial distribution and risk factors associated with human CE could help to prevent and control echinococcosis in China.

## 1. Introduction

Echinococcosis, also referred to as hydatid disease, is a zoonotic parasitic disease that seriously endangers the health of humans and imposes a heavy financial burden on sick families [1,2,3]. Human echinococcosis is caused by tapeworms of the genus Echinococcus [4,5]. To date, six forms of this tapeworm have been identified, and four of these represent a public health concern: *Echinococcus granulosus*, *E. multilocularis*, *E. vogeli* and *E. oligarthrus*, which cause cystic echinococcosis (CE), alveolar echinococcosis, polycystic echinococcosis and unicystic echinococcosis, respectively [6,7]. CE is one of the most common forms and is therefore of significant relevance to humans from a medical and public health point of view [8]. Data from the World Health Organization (WHO) shows that CE is distributed globally, except for Antarctica [9]. Worldwide, more than one million people are affected by echinococcosis at any one time [9].

China is one of the countries reporting the highest prevalence of human echinococcosis in the world, dominated by CE [10,11]. Echinococcosis is common in Western China and key areas in the Qinghai-Tibet plateau [12]. A previous study of the endemic status of echinococcosis [13] showed that a total of 10,790 cases of echinococcosis were reported in China between 2004 and 2008, and that 98.2% of patients with echinococcosis resided in China’s Western Xinjiang Uygur Autonomous Region, Inner Mongolia Autonomous Region, Ningxia Hui Autonomous Region, Sichuan, Qinghai, and Gansu provinces.

CE is widely distributed in Western China, but its spatial distribution, and the environmental factors driving its prevalence, are not clear. This study aimed to identify the geographical environment factors that affect the spatial distribution of human CE from a regional perspective. Numerous studies [14,15,16,17,18,19,20,21,22,23,24] have shown that human CE is closely related to natural, cultural environmental factors, including temperature, rainfall, elevation, vegetation, land use, education, economics, religious beliefs and professional factors; collectively, these factors are thought to underlie the epidemic transmission dynamics of human CE. Over recent years, several studies have evaluated the risk factors for human CE and the specific relationship between these factors and the prevalence of human CE in China. For example, Yang, Y.R. et al. [23] surveyed the impact of anthropogenic and natural environmental changes on Echinococcus transmission over the past 50 years in Ningxia of China, and they suggested that land use and micro-climate are important for the transmission cycles of CE. In another study, Yang, Y.R. et al. [15] surveyed 4773 individuals from 26 villages in Ningxia Hui Autonomous Region from 2002 to 2003 and revealed that potential risk factors included income and limited education. Yu, S.H. et al. [25] conducted an investigation in Jiuzhi County, Qinghai Province, and showed that Jiuzhi County is an important endemic area for human CE, and that risk factors included the Tibetan population, herdsmen, and Buddhist priests. Related research has already been conducted, but these studies have significant limitations. Only qualitative descriptive statistical analysis, which targeted biological factors related to the risk of human CE spreading at an individual level in a local area while rarely taking a quantitative approach to the relationship between environmental factors and the distribution of echinococcosis at a regional scale. Also, the geographical distribution of epidemic diseases has its specific regions. In some places, the disease is distributed and highly prevalent, while in some others, it is not and at low levels. This article addresses the geographical environment background cause of these phenomena, i.e., the significance of this study is to explain the ecological background (ecological driving force) of geographical distribution heterogeneity of human infection rate from a macro perspective (county-level). At the same time, since the distribution of human CE is unknown in all counties in seven western provinces and the prevalence data of echinococcosis are very scarce, leading to inconsistent data acquisition times, it is necessary and meaningful to make a risk prediction to prevent and control the spread of human CE in Western China. Therefore, the purpose of this paper was to determine the risk factors associated with human CE on a macro-scale (county-level) that covers seven provinces, not an individual level in a local area (one county or one village), and to gain a better understanding of the spatial distribution of human CE in Western China. 

Data arising from this study could thus be used to prevent and control the spread of human CE. In order to do this, we utilized remote sensing (RS) technologies with strong information acquisition capability. We also used geographic information system (GIS) technology to allow strong geographic spatial analysis and statistical methods which could help us to analyze risk-related factors and the spatial distribution of human CE [1,26]. Spatial statistical analyses have been widely applied in terms of spatial distribution estimation and simulation in other aspects of environmental science research [27]. Herein, these analytical techniques and methods were used to determine the risk factors associated with the spatial distribution of disease and to study the spatial distribution of human CE.

## 2. Materials and Methods 

### 2.1. Study Area

**Figure 1:**
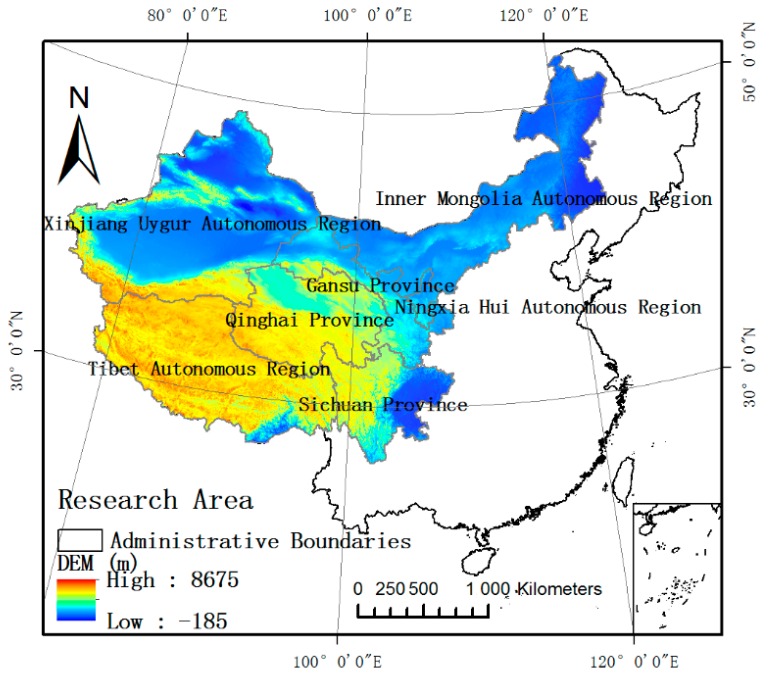
Map showing the research location. Note: DEM = digital elevation model.

The research area, shown in Figure 1, is located in Western China and covers an area of 5.64 million km^2^, accounting for 58.75% of the total area of China. Administratively, the research area is divided into seven provinces including Xinjiang Lygur Autonomous Region, Tibet Autonomous, Inner Mongolia Autonomous Region, Qinghai Province, Ningxia Hui Autonomous Region, Sichuan Province, and Gansu Province. These provinces are subdivided into 80 municipal administrative regions and 616 county-level administrative regions. In 2017, the permanent total population was approximately 170.96 million inhabitants, accounting for 12.5% of the total population of China [28].

### 2.2. Data Sources and Processing

#### 2.2.1. Prevalence of Human CE

Data relating to the prevalence of CE were mainly derived from previous scientific publications. We used “human cystic echinococcosis”, “cystic echinococcosis”, “human hydatid disease”, “*Echinococcus granulosus*” and “China” as key words to search PubMed, EBSCOhost, Excerpta Medica Database (Embase), the Cochrane Library, China Biomedical Literature Service System (SinoMed), WanFang Data, and China National Knowledge Infrastructure (CNKI) using the Google academic search engine. This search strategy was supplemented by manual retrieval and literature trace collection methods. Collectively this comprehensive strategy allowed us to acquire published data, at a county-level, from 2012 to 2018 in seven provinces of Western China. A total of 145 Chinese papers and 28 English papers were retrieved. These were downloaded, sorted, and relevant morbidity data were extracted. Ultimately, 33 articles and one survey report were used as our final valid reference (Reference S1). These papers featured prevalence data relating to 243 counties and were recorded in a GIS database; 63 counties were in Xinjiang, 15 counties in Gansu, 20 in Ningxia, 10 in Inner Mongolia, 72 in Tibet, 35 in Sichuan, and 28 in Qinghai. The date distribution of data sampling is mainly from 2012–2016, see Table 1. The data we obtained from different references were investigated using a consistent methodology. According to the People’s Republic of China Health Industry Standard—Diagnostic Criteria for Echinococcosis (WS257-2006), administrative villages were selected using a stratified cluster sampling method in counties, and residents were selected for B-mode ultrasound examination, supplemented by serological examination for suspected cases.

The prevalence rate in the county is defined as follows [29]:

(1) $$\;p=\frac{n}{N}\times 100\%\;$$

where, *p* is the prevalence rate, *n* is the number of patients detected, and *N* is the total number investigated.

#### 2.2.2. Natural Geographical Environmental Data

Data relating to a range of natural geographical factors were acquired, including climatological data, digital elevation model (DEM) data, normalized difference vegetation index (NDVI) data, land surface temperature (LST) data, and land use data. The time of all natural geographical environment data variables was consistent with the survey time of CE prevalence analyzed for each county. See Table 2 for more information.

• Climatological Data

Climatological data represented daily data (including mean temperature and precipitation) acquired from China’s 2400 meteorological stations between 2012 and 2016; these were obtained from the China Meteorological Data Sharing Service System [30]. The daily meteorological station data were interpolated for temperature and precipitation raster datasets of 500 m resolution by ANUSPLIN version 4.37 software (Hutchinson M F, Canberra, Australia). Annual mean temperature (Ta) and annual mean precipitation (Pa) data were obtained by averaging the daily temperature and precipitation raster datasets from 2012 to 2016.

• DEM Data

DEM can fully reflect geomorphological microfeatures and reveal the regional geomorphological space differentiation. Elevation data refers to ASTER GDEM V2 global digital elevation data, which is based on advanced spaceborne thermal emission and radiometer (ASTER) data developed jointly by the Ministry of Economy, Trade, and Industry (METI) of Japan and the United States National Aeronautics and Space Administration (NASA) [31].

• Normalized Difference Vegetation Index Data

NDVI is a quantitative indicator of whether the Earth’s surface is covered by vegetation and the characteristics of vegetation changes. MOD13Q1NDVI data (250 m resolution) were acquired from the U.S. Geological Survey [32] for the period between 2012 and 2016. MOD13Q1NDVI data required the MODIS Reprojection Tools (MRT) tool to allow formatting and projection conversion. In order to eliminate the effect of abnormal values, the NDVI data were synthesized by the maximum synthesis method (MVC), and the largest monthly NDVI image was used to characterize vegetation coverage. Then, the spring, summer, autumn, winter, and annual NDVI value were determined from 2012 to 2016.

• Land Surface Temperature Data

LST refers to the temperature of the ground. Surface temperature is a key factor in physical processes at both a regional and global scale and is also an important parameter with which to study the exchange of material and energy between the Earth’s surface and atmosphere. Land surface temperature data was used in a monthly synthetic (MODLT1M) product with a resolution of 1 km. The data set was provided by the International Scientific & Technical Data Mirror Site, Computer Network Information Center, Chinese Academy of Sciences [33]. We then determined the spring, summer, autumn, winter, and annual LST values for the period between 2012 and 2016.

• Land Use Data

Data relating to land use were provided by the Data Center for Resources and Environmental Sciences, Chinese Academy of Sciences (RESDC) [34]. The types of land use include cultivated land, forest land, grassland, water area, residential and unused land with six primary types and 25 secondary types.

Using the spatial analyst tools in ArcGIS 10.4 software (ESRI INC., Redlands, CA, USA), the annual mean temperature (Ta) data, annual average precipitation (Pa) data, DEM, NDVI, and LST was extracted by the county administrative region, and the mean value was calculated as an index. The proportion of arable land area, woodland area, grassland, water area, residential land area, and unused land area across the area of the whole county were extracted and calculated separately using a field calculator tool.

#### 2.2.3. Human Geographical Environment Data

Human geography factors included population density, religious beliefs, illiteracy rate, occupation, and gross domestic product. Data relating to population, religion, illiteracy, and occupation were obtained from the sixth census while Gross Domestic Product (GDP) data were acquired from the county-level statistical yearbook of the National Bureau of Statistics of the People’s Republic of China [35]. The time of all human geographical environment data variables was consistent with the survey time of human CE prevalence analyzed for each county. For more information, see Table 2.

• Population Density Data

Population density reflects the population concentration per unit area.

• Religious Beliefs Data

The western region of China mainly includes ethnic minorities such as Tibetan, Hui, and Mongolian. The relationship between religious belief and human echinococcosis can be reflected by the relationship between Tibetan population ratio, Mongolian population ratio, and Hui population ratio and the respective prevalence of human echinococcosis.

• Illiteracy Rate Data

The illiteracy rate reflects the cultural education level of the people within the study area.

• Occupation Data

The agricultural occupational population rate (AOPR) can reflect the composition of the professional population.

• Gross Domestic Product Data

GDP refers to the market value of all final products and services produced by the permanent units of a country or region within a certain period. GDP is a basic indicator of macroeconomic development.

These data were collated and stored in Microsoft Excel at a county level and the county name was identified as the unique identification field. Using the ‘Join’ function of ArcGIS 10.4, a data table was collated with the county vector administrative division property table and exported to the geospatial database.

### 2.3. Statistical Analysis and Risk Mapping

Based on the human CE spatial database table, 80% of the prevalence data were randomly sampled as modeling data to build the model and 20% of the prevalence data as testing data were compared with its predicted value. Multivariate linear regression analysis was used to identify significant environmental factors associated with the spatial distribution of human CE. Univariate correlation analyses were initially performed to test the effect of each variable on the distribution of human CE. Relationships were determined using correlation coefficients and *p* values. Variables with *p* values < 0.05 were defined as being statistically significant and were analyzed further. Relatively high co-linear variables were excluded by checking variance inflation factors (VIFs) and the correlation coefficient. VIFs > 10 were considered to have high co-linearity [36]. Logarithmic transformation was applied to transform prevalence data which did not initially conform with the normal distribution. Logarithmic transformation of original prevalence data was performed using the logarithmic function of the data conversion tool in SPSS 23.0 software (SPSS Inc., Chicago, IL, USA). Then, the transformed data was tested for normal distribution, and the transformed data was finally confirmed to conform to normal distribution. Variables showing statistical significance were selected and re-analyzed by multivariate linear regression to build a final model with a statistical significance level of *p* < 0.05. The goodness of fit of the model was evaluated using the root mean squared error (RMSE) and the adjusted R square value [37]. The adjusted R square value varies from 0 to 1; the closer to 1 the value is, the better the model is.

To predict the risks of human CE, a risk map was created using ArcGIS 10.4 software on the basis of the predictive model derived from multivariate linear regression analysis. The calculation of the predicted prevalence and risk mapping operations were carried out in ArcGIS 10.4 software. To begin, a new field named “predictive risk” was created in the previously built geographic database table which contained risk factors data for all counties that the model needed to enter. Once again, based on the predictive model and related risk factors data, the predicted prevalence of all counties in the western seven provinces was calculated using the field calculator function. The “Field calculator” has powerful batch-processing table data capabilities by entering model formulas and input data. Finally, a risk map was created based on the mapping function. As a carrier of information, maps can be used to visualize spatial data with spatial location, distribution characteristics, quality, and quantity.

## 3. Results

### 3.1. Spatial Distribution of Existing Human CE

**Figure 2:**
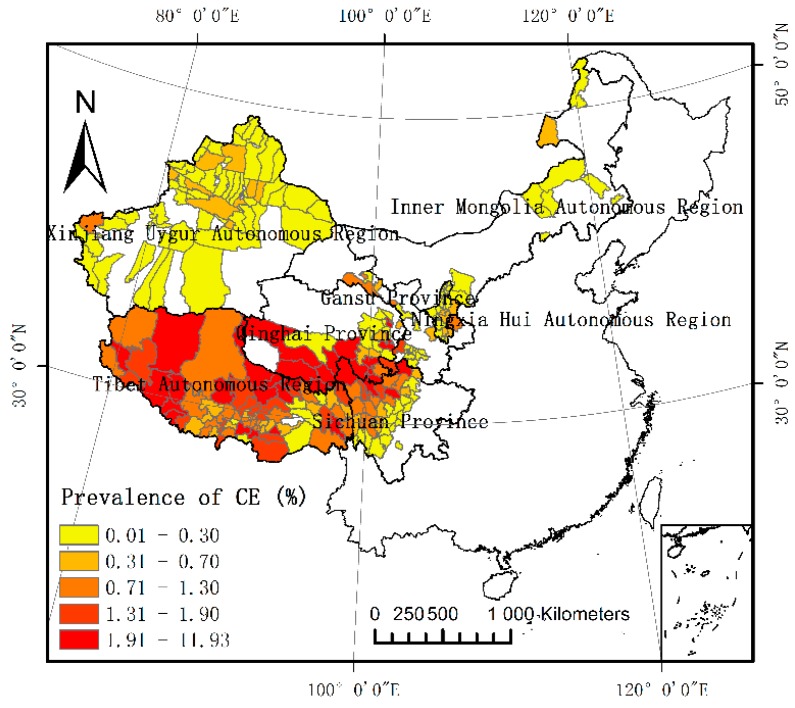
Spatial distribution of human cystic echinococcosis (CE) in Western China.

The spatial distribution of the prevalence of human CE in seven provinces of Western China is shown in Figure 2. This map shows that areas with a high prevalence of the disease are concentrated in the connected regions of Qinghai, Gansu, and Sichuan and in most of Tibet. The counties with the highest prevalence rates are Dari, Zuogong, Chengduo, Shiqu, and Cuomei with prevalences of 11.93%, 7.48%, 7.41%, 7.16% and 7.13%, respectively. The area with the lowest prevalence was Qumalai county (0.01%).

### 3.2. Univariate Analysis and Multivariate Analysis

The results of our univariate analysis and multivariate analysis (coefficient and significance) are shown in Table 3. After univariate analysis, population density, Tibetan population ratio, Mongolian population ratio, Hui population ratio, illiteracy rate, AOPR, GDP, annual mean precipitation, annual mean temperature, DEM, NDVI (Winter), LST (Spring), LST (Summer), LST (Autumn), LST (Winter), LST (Annual average), arable area ratio, grassland area ratio, and residential area ratio indices were found to be highly correlated variables and input into a subsequent multiple regression analysis model. Forward multiple regression analysis indicated that GDP, LST (Spring), grassland area ratio, and Tibetan population ratio represented significant risk factors for human CE. Grassland area ratio and Tibetan population ratio showed significant positive correlations while GDP and LST (Spring) showed significant negative correlations. The adjusted R square value of the multivariate linear regression equation was 0.71 (*p* < 0.01) and the RMSE was 0.14, which meant that the multiple regression analysis model could effectively fit the relationship between the environmental factors and human CE. The relationship model between the prevalence of human CE and related geographical environment factors could therefore be expressed as follows:

(2)  Y=Exp(−2.20−0.01×GDP−0.05×LST (Spring)+1.46×Grassland area ratio+1.56×Tibetan population ratio) 

### 3.3. Predicted Risk Distribution of Human CE

**Figure 3:**
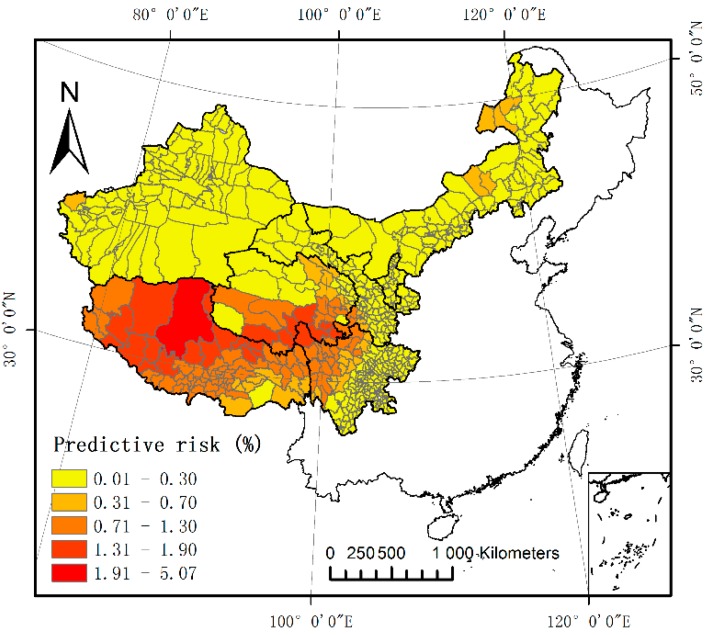
Predictive risk map of human cystic echinococcosis (CE) in Western China.

Based on the final multiple regression analysis (Equation (2)), a predictive risk map of human CE was created for Western China, as shown in Figure 3. We divided the predicted prevalence into five grades and defined counties with a prevalence rate of 0.71–5.07 as high-risk areas. High-risk areas were mainly located in the south of Qinghai, the northwest of Sichuan, and most of the Tibet Autonomous Region. There were 102 counties in high-risk areas, accounting for 16.60% of the total counties.

## 4. Discussion

Our present study identified that the prevalence of human CE is closely related to natural environment and cultural factors, such as precipitation, temperature, NDVI, DEM, LST, land use, occupation, and other factors. However, these factors, which relate to the immediate host of human CE and definitive host habitats, are complicated and exert combined effects, especially in the vast western region of China. Typical factors were selected and quantified using multiple linear regression model analysis (Equation (2)). Our analysis identified grassland area ratio and Tibetan population ratio as significant and positively correlated independent variables, while GDP and LST (Spring) were significant but negatively correlated independent variables. Finally, a predictive risk map of CE was created for Western China.

LST (Spring) was negatively correlated with human CE. Land surface temperature reflects a temperature variable [38]. In particular, Echinococcus eggs are sensitive to high land surface temperature [39], and the environment can influence echinococcosis by affecting the maturation and survival time of the eggs [16,40,41]. In theory, areas with low surface temperatures could be more popular for echinococcosis. A recent study [42] showed that deep-freezing at −18 °C to −20 °C does not kill eggs of E. multilocularis, and they can only be killed at −70 °C to −80 °C. Further research by J. Eckert [43] showed that land surface temperature has a negative association and is involved in the transmission of CE in Rangtang County of the Tibetan plateau. The western region, due to its high altitude, has a typical plateau mountain climate, and the surface temperature remains low for years; consequently, this is a very suitable area for echinococcosis. LST (Spring) is statistically significant in multivariate regression analysis. However, multivariate regression analysis does not show that LST in autumn and winter is not related to the prevalence of human CE. A possible reason for the preliminary analysis is that LSTs (Spring) have a greater impact on the prevalence of echinococcosis than those in autumn and winter. Other possible reasons are as follows. Some studies [44] have shown that early winter and early spring are the main infectious seasons for dogs. The larvae develop and reproduce in the dog’s gut, and large numbers of eggs are excreted through feces. Low surface temperatures in spring can affect the survival time of the eggs, which in turn may increase the probability of human infection through the ingestion of water infected with the eggs.

In our study, “Tibetan population ratio” was associated with an increased likelihood of human infection. The detection rate of Tibetan human CE was previously shown to be the highest among different ethnic groups [45,46]. This may be associated with the local ethnic customs and religious beliefs. Tibetans generally like to keep a large number of dogs to guard livestock and property, and they are therefore often in close contact with their dogs [47,48]. Furthermore, the majority of Tibetans are Buddhists, who claim to be non-violent and ban the killing of any animal, including dogs [49]. This behavior leads to a large number of stray dogs, which tend to congregate around temples and villages, because they are fed by monks and herders [46,50]. Dogs also prey upon small mammals near pastures, and these dogs are usually fed by herders with the heart and lungs of cattle and sheep during the slaughtering season [12,47,51]. These facts are important because dogs are the main host of echinococcosis and have been identified as the primary source of human CE [47,52]. Therefore, the Tibetan population ratio plays an important role in the transmission of human CE.

Grassland area ratio represents the grassland coverage density of the environment and was positively correlated with human CE. Grassland vegetation cover directly affects the distribution of intermediate hosts and plays an important ecological role in the spread of the disease [53,54]. Grassland is one of the habitat conditions required for the growth and reproduction of intermediate hosts, such as horse, cattle, and sheep [23,55]. West China is an important agricultural and pastoral area with vast grasslands, and farmers and herdsmen in this area keep a large number of cattle and sheep. As a result, a large number of cattle and sheep eat the grass, water, and soil infected with Echinococcus eggs. The larvae then develop in the guts of the intermediate host cattle and sheep, and dogs become infected by eating the guts of cattle and sheep. The definitive host dog excretes large amounts of eggs through feces, and eventually, humans become infected by ingesting food such as water infected with the eggs. This chain of events makes it possible to maintain disease transmission between grass and hosts.

GDP was also shown to have a negative effect on human CE. GDP is a common socio-economic indicator that reflects the regional economic development and household income. The underdeveloped economy is one of the main causes of the epidemic of human CE [24,56]. In underdeveloped rural areas of Western China, people generally do not have the awareness to accept a medical examination before the illness [57]. The backwardness of education, lack of knowledge, and poor hygiene habits caused by economic backwardness make people vulnerable to echinococcosis. At the same time, the economic backwardness leads to a lack of medical resources, low family income, and inconvenient transportation, so that patients cannot go to the hospital in sufficient time. Indeed, some studies [45,58] have shown that an underdeveloped economy and low income are important factors in the spread of echinococcosis. Furthermore, Yu Rong Yang [3] showed that household income levels strongly influenced the choice of health care provider, and despite the higher quality and more efficient diagnosis and treatment available in the city, residents preferred to seek health care in local county hospitals in Ningxia of China. A more recent study [59] showed that echinococcosis is closely related to economic development and residents’ income, and that this can be one of the major factors of disease transmission.

Finally, we simulated and predicted a risk map of human CE in Western China after determining the geographical environment factors that affected disease transmission. As shown in the risk prediction map, the areas with a high prevalence were mainly concentrated in the southern part of Qinghai, the northwest of Sichuan, and most of the Tibet Autonomous Region. The high-risk areas were mainly located in the Qinghai-Tibet plateau region of China; this is an area associated with relatively low surface temperatures and is also the main region of the Tibetan population, with a large number of alpine grasslands [12,60]. Further research by Wang showed that the Qinghai-Tibet Plateau is the most endemic area in China [44]; a subsequent study [61] showed that the Eastern Tibetan Plateau was highly endemic for CE. In another study, Budke et al. [52] noted that the Tibetan plateau of Western China showed a very high prevalence of human CE. Our new risk prediction map can reflect the distribution of human CE in Western China.

This study was limited by an inconsistent survey time of the prevalence of human CE across different regions. However, Human CE has a complex propagation path and an asymptomatic incubation period, which can be sustained for years until the parasite larvae evolve and trigger clinical signs. Thus, it is feasible to analyze the relationship between prevalence and environmental factors over several years. However, we also call for continuous systematic echinococcosis monitoring that will support future studies of the spatiotemporal distribution of echinococcosis and the factors that influence the prevalence of this disease.

## 5. Conclusions

This study investigated the prevalence and geographical environment factors associated with human CE in Western China where the prevention and control of this disease is a most urgent task. Our study provided an efficient approach with which to identify environmental determinants using remote sensing, GIS spatial analysis, and multiple regression analysis modeling at the county-level. Spatial analysis showed that the disease was widely distributed and prevalent in Western China. Our study indicates that GDP, grassland area ratio, Tibetan population ratio, and LST (Spring) were significant risk factors and can contribute to the spread of the disease. The predictive risk map shows the areas with high risk for human CE infection. Echinococcosis remains a serious public health problem, and the prevention and control of echinococcosis is a priority in Western China. Some of our suggestions are as follows. (1) Our findings can be submitted to the Ministry of Agriculture and Rural Affairs and the National Health Commission of the People’s Republic of China to help government agencies understand the current epidemic distribution of echinococcosis in Western China, and can also serve as a practical guide for health workers in local centers for disease control and prevention to implement enhanced surveillance in key endemic areas to reduce the number of future human infections. (2) We suggest that the government should actively carry out health education, popularize the knowledge of echinococcosis and make the Tibetan people form good health behaviors. The government is advised to control the number of infectious dogs, especially wild dogs. (3) The western region is mainly formed of agricultural and pastoral areas, with large numbers of cattle and sheep. The government should strengthen the management of animal slaughter, strictly implement the system of food hygiene inspection and animal quarantine, and guide people not to feed dogs with untreated diseased organs. (4) The government should speed up the development of the economy in the west, especially in remote rural areas and effectively improve people’s medical conditions. (5) It is recommended that the government strengthen drug repellent measures for dogs during the main infectious season of dogs, and that the government should guide people to develop a good habit of drinking clean water. Our findings and suggestions are of great significance to solving the national public health security, reducing economic losses for the country, and reducing the burden for many families in the future.

## Figures and Tables

**Table 1:** Date of data survey.

Survey Date	2012	2013	2014	2015	2016
Number of counties investigated	163	0	3	1	76

**Table 2:** Geographical environment data.

Data Categories	Name	Source	Unit
Climatological data	Annual mean temperature	China Meteorological Data Sharing Service System	°C
	Annual average precipitation		mm
DEM data	DEM	NASA	m
NDVI data	NDVI (Spring)	U.S. Geological Survey	-
	NDVI (Summer)		-
	NDVI (Autumn)		-
	NDVI (Winter)		-
	NDVI (Annual average)		-
LST data	LST (Spring)	International Scientific & Technical Data Mirror Site, Computer Network Information Center, Chinese Academy of Sciences	°C
	LST (Summer)		°C
	LST (Autumn)		°C
	LST (Winter)		°C
	LST (Annual average)		°C
Land use data	Arable area ratio	Data Center for Resources and Environmental Sciences, Chinese Academy of Sciences	-
	Woodland area ratio		-
	Grassland area ratio		-
	Water area ratio		-
	Residential area ratio		-
	Unused area ratio		-
Population density data	Population density	National Bureau of Statistics of the People’s Republic of China	people/km^2^
Religious beliefs data	Tibetan population ratio		-
	Mongolian population ratio		-
	Hui population ratio		-
Illiteracy rate data	Illiteracy rate		-
Occupation data	AOPR		-
GDP data	GDP		10^6^ million RMB yuan

Note: DEM = digital elevation model; NDVI = normalized difference vegetation index; LST = land surface temperature; AOPR = agricultural occupational population rate; GDP = Gross Domestic Product; NASA = United States National Aeronautics and Space Administration; RMB yuan refers to the Chinese currency unit.

**Table 3:** Results arising from the multivariate linear regression analysis.

Factors	Univariate Analysis		Multivariate Analysis	
	Coefficient	*p* Value	Coefficient	*p* Value
Population density	−0.50 **	0.00		
Tibetan population ratio	0.63 **	0.00	1.56	0.00
Mongolian population ratio	−0.44 **	0.00		
Hui population ratio	−0.44 **	0.00		
Illiteracy rate	0.58 **	0.00		
AOPR	0.23 **	0.00		
GDP	−0.56 **	0.00	−0.01	0.00
Annual mean precipitation	0.31 **	0.00		
Annual mean temperature	−0.54 **	0.00		
DEM	0.66	0.00		
NDVI (Spring)	−0.08	0.27		
NDVI (Summer)	−0.01	0.85		
NDVI (Autumn)	0.00	0.98		
NDVI (Winter)	0.25 **	0.00		
NDVI (Annual average)	0.03	0.71		
LST (Spring)	−0.50 **	0.00	−0.05	0.01
LST (Summer)	−0.51 **	0.00		
LST (Autumn)	−0.51 **	0.00		
LST (Winter)	−0.44 **	0.00		
LST (Annual average)	−0.55 **	0.00		
Arable area ratio	−0.58 **	0.00		
Woodland area ratio	−0.10	0.18		
Grassland area ratio	0.50 **	0.00	1.46	0.00
Water area ratio	0.00	0.95		
Residential area ratio	−0.50 **	0.00		
Unused area ratio	−0.07	0.36		

Note: AOPR = agricultural occupational population rate; GDP = Gross Domestic Product; DEM = digital elevation model; NDVI = normalized difference vegetation index; LST = land surface temperature; ** indicates a significant correlation at the 0.01 level (2-tailed).

## Supplementary Materials

The following are available online at http://www.mdpi.com/1660-4601/15/8/1729/s1, Reference S1: References for data on the prevalence of human CE.
