# Supplementary material for: Geographical Environment Factors and Risk Mapping of Human Cystic Echinococcosis in Western China
Source: Int J Environ Res Public Health. 2018 Aug 12;15(8):1729. doi: 10.3390/ijerph15081729 (PMC6121593; doi:10.3390/ijerph15081729)
Supplement: Supplementary file 1 [file ijerph-15-01729-s001.pdf]

甘肃省(Gansu Province)

高台县(Gaotai County)[1]
玛曲县(Maqu County)[2]
会宁县(Huining County)[3,4]
肃南裕固族自治县(Sunan Yugur Autonomous County)[2]
天祝藏族自治县(Bairi Tibetan Autonomous County)[5]
漳县(Zhang County)[6]
环县(Huan County)[7]
合作市(Hezuo City)[8]
临潭县(Lintan County)[8]
卓尼县(Zhuoni County)[8]
夏河县(Xiahe County)[2,7]
迭部县(Têwo County)[8]
甘州区(Ganzhou Distict)[9]
皋兰县(Gaolan County)[2]
岷县(Min County)[2]

青海省（Qinghai Province）

共和县(Gonghe County)[7]
同德县(Tongde County)[10]
贵德县(Guide County)[10]
贵南县(Guinan County)[10]
兴海县(Xinghai County)[10]
久治县(Jiuzhi County)[11]
达日县(Dari County)[11]
班玛县(Banma County)[11]
甘德县(Gande County)[11]
玛多县(Maduo County)[11-13]
玛沁县(Maqin County)[11]
曲麻莱县(Qumalai County)[14]
泽库县(Zeku County)[15]
河南蒙古族自治县(Henan Mongol Autonomous County)[15]
玉树县(Yushu County)[7,14]
杂多县(Zaduo County)[14]
治多县(Zhiduo County)[12-14]
囊谦县(Niangqian County) [14]
大通回族土族自治县(Datong Hui and Tu Autonomous County)[16]
湟源县(Huangyuan County)[7]
乐都县(Ledu County)[17]
湟中县(Huangzhong County)[18]
城东区(Chengdong District)[18]
城西区(Chengxi District)[18]
城中区(Chengzhong District)[18]
城北区(Chengbei District)[18]
海晏县(Haiyan County)[19]
称多县(Chengduo County)[20]

宁夏回族自治区（Ningxia Hui Autonomous Region）

原州区(Yuanzhou District)[21]

同心县(Tongxin County)[21]
盐池县(Yanchi County)[21]
彭阳县(Pengyang County)[21]
红寺堡区(Hongbao Si District)[21]
中宁县(Zhongning County)[21,22]
隆德县(Longde County)[21]
西吉县(Xiji County)[21]
西夏区(Xixia District)[21]
青铜峡市(Qingtong Xia City)[21]
利通区(Litong District)[21]
沙坡头区(Shapo Tou District)[21]
贺兰县(Helan County)[21]
海原县(Haiyuan County)[21]
永宁县(Yongning County)[21]
惠农区(Huinong District)[21]
泾源县(Jingyuan County)[21]
大武口区(Dawu Kou District)[21]
灵武市(Lingwu City)[21]
平罗县(Pingluo County)[21]

新疆维吾尔自治区(Xinjiang Lygur Autonomous Region)

新市区(Xinshi District)[23]
乌恰县(Ulugqat County)[23]
水磨沟区(Shuimogou Distric)[23]
托里县(Toli County)[23]
和布克赛尔蒙古自治县(Hoboksar Mongol Autonomous County)[23]
吉木萨尔县(Jimsar County)[23]
尼勒克县(Nilka County)[23]
霍城县(Korgas County)[23]
阜康市(Fukang City)[23]
博湖县(Bagrax County)[23]
乌鲁木齐县(Urumqi County)[23]
和静县(Hejing County)[23]
阿图什市(Artux City)[23]
焉耆回族自治县(Yanji Hui Autonomous County)[23]
博乐市(Bortala City)[23]
巴里坤哈萨克自治县(BarkolKazak Autonomous County)[23]
巩留县(Tokkuztara County)[23]
裕民县(Qagantokay County)[23]
新源县(Kunes County)[23]
阿克陶县(Akto County)[23]
吐鲁番市(Turpan City)[23]
策勒县(Qira County)[23]
且末县(Qarqan] County)[23,24]
额敏县(Dorbiljin County)[23]
吉木乃县(Jemnay County)[23]
塔什库尔干塔吉克自治县(Taxkorgan Tajik Autonomous County)[23]
乌尔禾区(Urhe District)[23]
福海县(Burultokay County)[23]
乌苏市(Usu City)[23]
奇台县(Qitai County)[23]

阿合奇县(Akqi County)[23]
伊吾县(Araturuk County)[23]
轮台县(Bugur County)[23]
墨玉县(Karakax County)[23]
米东区(Midong District)[23]
达坂城区(Dabancheng District)[23]
塔城市(Qoqek City)[23]
和硕县(Hoxud County)[23]
木垒哈萨克自治县(Mori Kazak Autonomous County)[23]
呼图壁县(Hutubi County)[23]
温泉县(Araxang County)[23]
托克逊县(Toksun County)[23]
伊宁市(Gulja City)[23]
富蕴县(Koktokay County)[23]
伊宁县(Gulja County)[23]
温宿县(Onsu County)[23]
哈巴河县(Kaba County)[23]
特克斯县(Tekes County)[23]
叶城县(Kagilik County)[23]
玛纳斯县(Manas County)[23]
察布查尔锡伯自治县(Qapqal Xibe Autonomous County)[23]
沙湾县(Shawan County)[23]
阿勒泰市(Altay City)[23]
布尔津县(Burqin County)[23]
哈密市(Kumul City)[23]
克拉玛依区(Karamay District)[23]
尉犁县(Lopnur County)[23]
沙雅县(Xayar County)[23]
于田县(Keriya County)[23]
青河县(Qinggil County)[23]
昌吉市(Changji City)[23]
精河县(Jing County)[23]
阿克苏市(Aksu City)[23]

四川 (Sichuan Province)

阿坝县(Aba County)[25]
巴塘县(Batang County)[25]
白玉县(Baiyu County)[25]
宝兴县(Baoxing County)[25]
丹巴县(Danba County)[25]
道孚县(Daofu County)[25]
稻城县(Daocheng County)[25]
德格县(Dege County)[25]
得荣县(Derong County)[25]
甘孜县(Ganzi County)[25]
黑水县(Heishui County)[25]
红原县(Hongyuan County)[25]
金川县(Jinchuan County)[25]
九龙县(Jiulong County)[25]
九寨沟县(Jiuzha Gou County)[25]
康定县(Kangding County)[25]

理塘县(Litang County)[25]
理县(Li County)[25]
泸定县(Luding County)[25]
炉霍县(Luhuo County)[25]
马尔康县(Maer Kang County)[25]
茂县(Mao County)[25]
木里藏族自治县(Muli Tibetan Autonomous County)[25]
壤塘县(Rangtang County)[25]
若尔盖县(Ruoer Gai County)[25]
色达县(Seda County)[25]
石渠县(Shiqu County)[25]
松潘县(Songpan County)[25]
天全县(Tianquan County)[25]
汶川县(Wenchuan County)[25]
乡城县(Xiangcheng County)[25]
小金县(Xiaojin County)[25]
新龙县(Xinlong County)[25]
雅江县(Yajiang County)[25]
越西县(Yuexi County)[25]

内蒙古自治区（Inner Mongolia Autonomous Region）

锡林浩特市(Xilinhot City)[26]
扎鲁特旗(Jarud Banner)[7,26]
阿巴嘎旗(Abag Banner)[7,26]
额尔古纳市(Ergun City)[26]
西乌珠穆沁旗(Xiwuzhumuqin Banner) [26]
东乌珠穆沁旗(East Ujimqin Banner)[26]
太仆寺旗(Taipusi Banner)[26]
科尔沁区(Keerqin District)[26]
鄂托克前旗(Etuokeqian Banner)[26]
鄂托克旗(Etuoke Banner)[26]
新巴尔虎右旗(Xin Barag Youqi)[26]

西藏自治区（Tibet Autonomous）

波密县(Bomi County)[27]
察隅县(Chayu County)[27]
工布江达县(Gongbu Jiangda County)[27]
朗县(Lang County)[27]
米林县(Milin County)[27]
墨脱县(Motuo County)[27]
措勤县(Cuoqin County)[28]
改则县(Gaize County)[28]
噶尔县(Geer County)[28]
革吉县(Geji County)[28]
普兰县(Pulan County)[28]
札达县(Zhada County)[28]
日土县(Ritu County)[28]
措美县(Cuomei County)[29]
安多县(Amdo County)[30]
巴青县(Baqen County)[30]
班戈县(Bange County)[30]

比如县(Biru County)[30]
嘉黎县(Jiali County)[30]
那曲县(Nagqu County)[30]
尼玛县(Nima County)[30]
聂荣县(NieRong County)[30]
申扎县(Shenzha County)[30]
索县(Suo County)[30]
浪卡子县(Langkazi County)[31]
隆子县(Longzi County)[31]
错那县(Cuona County)[31]
扎囊县(Zhanang County)[31]
贡嘎县(Gongga County)[31]
乃东区(Naidong District)[31]
琼结县(Qiongjie County)[31]
加查县(Jiacha County)[31]
桑日县(Sangri County)[31]
曲松县(Qusong County)[31]
洛扎县(Luozha County)[31]
桑珠孜区(Sangzhu Zi County)[32]
南木林县(Namling County)[32]
江孜县(Gyangze County)[32]
定日县(Tingri County)[32]
萨迦县(Sakya County)[32]
拉孜县(Lhaze County)[32]
昂仁县(Ngamring County)[32]
谢通门县(Xaitongmoin County)[32]
白朗县(Bainang County)[32]
仁布县(Rinbung County)[32]
康马县(kangmar County)[32]
定结县(Dingggye County)[32]
仲巴县(Zhongba County)[32]
亚东县(Yadong County)[32]
吉隆县(Gyirong County)[32]
聂拉木县(Nyalam County)[32]
萨嘎县(Saga County)[32]
岗巴县(Gamba County)[32]
当雄县(Damxung County)[33]
墨竹工卡县(Maizhokunggar County)[33]
林周县(Lhünzhub County)[33]
尼木县(Nyêmo County)[33]
达孜县(Dagzê County)[33]
城关区(Chengguan District)[33]
堆龙德庆区(Doilungdegen District)[33]
曲水县(Qüxü County)[33]
卡若区(Karuo District)[34]
江达县(Jangda County)[34]
贡觉县(Gongjue County)[34]
类乌齐县(Leiwuqi County)[34]
丁青县(Dingqing County)[34]
察雅县(Chaya County)[34]
八宿县(Basu County)[34]

左贡县(Zuogong County)[34]
芒康县(Mangkang County)[34]
洛隆县(Luolong County)[34]
边坝县(Bianba County)[34]

参考文献（References）

1. 蒲秀华; 陈睿; 康中北. 2012 年甘肃省高台县包虫病流行情况调查报告. 疾病预防控制通报 2013, 44-44.
2. 余大为; 丁国武; 侯言东; 冯宇; 李凡. 甘肃省细粒棘球蚴病聚类分析. 中国寄生虫学与寄生虫病杂志 2015, 33, 280-282.(Yu, D.W.; Ding, G.W.; Hou, Y.D.; Feng, Y.; Li, F. [clustering analysis of hydatid disease in gansu province]. Zhongguo ji sheng chong xue yu ji sheng chong bing za zhi = Chinese journal of parasitology & parasitic diseases 2015, 33, 281.)
3. 何昕; 宋刊芳; 巩转萍; 吉文博; 董志兵; 张永强; 李雪玲; 程永莲. 甘肃省会宁县包虫病防治现状调查. 疾病预防控制通报 2016, 39-41.
4. 何斌. 甘肃省会宁县包虫病流行情况调查. 中国媒介生物学及控制杂志 2013, 24, 177-177.(Bin, H.E. Investigation of prevalence of echinococcosis in huining county, gansu province, china. 2013.）
5. 买彩红; 史万贵; 王淑芳. 天祝县人畜包虫病防治调查及分析. 兽医导刊 2016, 5-6.
6. 马晓斌; 唐丹华; 裴红; 王爱宏. 甘肃省漳县包虫病流行病学调查. 疾病预防控制通报 2016, 35-37.(Xiao-Bin, M.A.; Tang, D.H.; Pei, H.; Wang, A.H. Epidemiologic survey of hydatid disease in zhang county,gansu province. Bulletin of Disease Control & Prevention 2016.）
7. 伍卫平; 王虎; 王谦; 周晓农; 王立英; 郑灿军; 曹建平; 肖宁; 王莹; 朱曜宇. 2012-2016年中国棘球蚴病抽样调查分析. 中国寄生虫学与寄生虫病杂志 2018.(Wei-Ping, W.U.; Wang, H.; Wang, Q.; Zhou, X.N.; Wang, L.Y.; Zheng, C.J.; Cao, J.P.; Xiao, N.; Wang, Y.; Zhu, Y.Y. A nationwide sampling survey on echinococcosis in china during 2012-2016. Chinese Journal of Parasitology & Parasitic Diseases 2018.)
8. 尚文杰; 张澍文. 甘南藏族自治州包虫病防控效果分析. 疾病预防控制通报 2018.(Shang, W.J.; Zhang, S.W. Effective analysis of prevention and control of echinococcosis in gannan tibetan autonomous prefecture. Bulletin of Disease Control & Prevention 2018.)
9. 刘晓梅. 张掖市甘州区 2015 年包虫病流行情况调查分析. 国外医学医学地理分册 2017, 38, 35-37.(Liu, X. Prevalence of hydatid disease in ganzhou district in 2015. Foreign Medical Sciences 2017.)
10. 蔡辉霞; 王虎; 韩秀敏; 马霄; 张静宵; 刘玉芳; 王永顺; 雷雯; 王威; 刘培运. 青海省海南藏族自治州棘球蚴病流行现状调查. 中国病原生物学杂志 2016, 1022-1025.
11. 马霄; 王虎; 张静宵; 王永顺; 程时磊; 刘玉芳; 马俊英; 蔡辉霞; 刘培运; 韩秀敏. 青海省果洛藏族自治州棘球蚴病和棘球绦虫病流行情况调查. 中国寄生虫学与寄生虫病杂志 2017, 35, 366-370.(Xiao, M.A.; Wang, H.; Zhang, J.X.; Wang, Y.S.; Cheng, S.L.; Liu, Y.F.; Jun-Ying, M.A.; Cai, H.X.; Liu, P.Y.; Han, X.M. Epidemiological investigation on hydatid disease/echinococcosis in guoluo tibetan autonomous prefecture in qinghai province. Chinese Journal of Parasitology & Parasitic Diseases 2017.)
12. 房琦; 伍卫平; 王立英; 曾祥嫚. 空间插值法在人群包虫病患病率预测中的应用. 中国病原生物学杂志 2014.(Fang, Q.; Wei-Ping, W.U.; Wang, L.Y.; Zeng, X.M. Use of spatial interpolation to predict the prevalence of hydatidosis. Journal of Pathogen Biology 2014, 9, 203-206.)
13. 房琦. 青藏高原地区棘球蚴病流行现状及影响因素分析. 中国疾病预防控制中心, 2014.
14. 程时磊; 王虎; 马霄; 张静宵; 刘玉芳; 蔡辉霞; 刘培运; 马俊英; 何多龙; 吴献洪. 2012 年青海省玉树藏族自治州棘球蚴病流行调查. 中国寄生虫学与寄生虫病杂志 2016, 34, 547-551.(Cheng, S.L.;

Wang, H.; Xiao, M.A.; Zhang, J.X.; Liu, Y.F.; Cai, H.X.; Liu, P.Y.; Jun-Ying, M.A.; Duo-Long, H.E.; Xian-Hong, W.U. An epidemiological survey on echinococcosis in yushu prefecture of qinghai province. Chinese Journal of Parasitology & Parasitic Diseases 2016.)
15. 马霄; 王虎; 程时磊; 张静宵; 王永顺; 刘玉芳; 马俊英; 蔡辉霞; 刘培运; 韩秀敏. 青海省黄南藏族自治州棘球蚴病流行情况调查. 中国寄生虫学与寄生虫病杂志 2017, 35, 512-514.(Xiao, M.A.; Wang, H.; Cheng, S.L.; Zhang, J.X.; Wang, Y.S.; Liu, Y.F.; Jun-Ying, M.A.; Cai, H.X.; Liu, P.Y.; Han, X.M. Epidemiological investigation on echinococcosis in huangnan tibetan autonomous prefecture of qinghai province. Chinese Journal of Parasitology & Parasitic Diseases 2017.)
16. 赵明奎; 闫立娟; 任更生. 青海省大通县 2012 年包虫病流行病学调查报告. 医学动物防制 2014.
17. 盛永华. 青海省乐都县包虫病感染现状调查. 青海医药杂志 2013, ;, 72-73.
18. 马小丽. 2013 年西宁市包虫病流行情况调查. 青海医药杂志 2014, 72-74.
19. 铁富萍; 才仁卓玛. 青海海晏人畜间包虫病的流行病学调查. 中国兽医杂志 2015, 51, 49-50.
20. 丁天龙. 青海省玉树州称多县包虫病流行病学现状分析. 青海大学, 2016.
21. 赵建华; 吴向林; 马荣; 付益仁; 冯运灵. 宁夏 2012 年棘球蚴病流行现况调查分析. 宁夏医学杂志 2014, 36, 314-316.(Zhao, J.; Xianglin, W.U.; Rong, M.A.; Yiren, F.U.; Feng, Y. Analysis of hydatid disease prevalence survey in ningxia in 2012. Ningxia Medical Journal 2014.)
22. 吴昊; 张振华; 张向国. 中宁县人群包虫病学现况调查分析. 宁夏医学杂志 2017, 39, 659-661.
23. 买买提江; 吾买尔; 阿迪力; 司马义; 伊斯拉音; 乌斯曼; 亚里昆; 买买提依明; 侯岩岩; 肖宁. 2012 年新疆维吾尔自治区人群棘球蚴病流行病学调查. 中国寄生虫学与寄生虫病杂志 2016, 34, 249-254.
24. 冀清萍; 薛成; 戴新安. 且末县 2012 年包虫病流行情况调查分析. 中外健康文摘 2013, 161-161.
25. Https://wenku.Baidu.Com/view/7c10ce1a941ea76e59fa04ce.Html?From=search.
26. 宋壮志; 郝慧霞; 姜晓峰; 卢爱桃; 南晓伟. 内蒙古自治区包虫病流行现况调查. 首都公共卫生 2017, 11, 53-55.
27. 王栋民; 何瑞峰; 贡桑曲珍; 肖丹; 索郎旺杰; 雪莲; 边巴卓玛; 李景中. 林芝市棘球蚴病流行情况. 中国寄生虫学与寄生虫病杂志 2018.(Wang, D.M.; Rui-Feng, H.E.; Qu-Zhen, G.; Xiao, D.; Wang-Jie, S.; Xue, L.; Zhuo-Ma, B.; Jing-Zhong, L.I. Prevalence of echinococcosis in nyingchi city. Chinese Journal of Parasitology & Parasitic Diseases 2018.)
28. 肖丹; 伍卫平; 雪莲; 贡桑曲珍; 次仁拉姆; 边巴卓玛; 王栋民. 阿里地区棘球蚴病流行现状. 中国寄生虫学与寄生虫病杂志 2018.(Xiao, D.; Wei-Ping, W.U.; Xue, L.; Qu-Zhen, G.; La-Mu, C.; Zhuo-Ma, B.; Wang, D.M. Prevalence of hydatid disease in ali prefecture. Chinese Journal of Parasitology & Parasitic Diseases 2018.)
29. 贡桑曲珍; 王立英; 牛彦麟; 白玛央金; 次仁拉姆; 肖丹; 王栋民; 李斌. 西藏自治区人群棘球蚴病空间分布特征分析. 中国病原生物学杂志 2018.
30. 旦珍旺久; 薛垂召; 贡桑曲珍; 艾佳佳; 罗钊辉; 旦增曲珍; 魏小刚; 郑灿军. 那曲地区棘球蚴病流行现状分析. 中国寄生虫学与寄生虫病杂志 2018.
31. 白玛央金; 伍卫平; 何瑞峰; 贡桑曲珍; 康珠益西; 索朗旺杰; 李斌. 山南市棘球蚴病流行现状调查. 中国寄生虫学与寄生虫病杂志 2018.(Yang-Jin, B.; Wei-Ping, W.U.; Rui-Feng, H.E.; Qu-Zhen, G.; Yi-Xi, K.; Wang-Jie, S.; Bin, L.I. Prevalence of echinococcosis in shannan city. Chinese Journal of Parasitology & Parasitic Diseases 2018.)
32. 边巴卓玛; 李斌; 陈伟奇; 王栋民; 肖丹; 边巴; 贡桑曲珍. 日喀则市棘球蚴病流行现状分析. 中国寄生虫学与寄生虫病杂志 2018.(Zhuo-Ma, B.; Bin, L.I.; Chen, W.Q.; Wang, D.M.; Xiao, D.; Bian, B.; Qu-Zhen, G. Current prevalence of echinococcosis in shigatse city. Chinese Journal of Parasitology & Parasitic Diseases 2018.)

33. 次仁拉姆; 严信留; 旦珍旺久; 龙章佑; 旦增曲珍; 艾佳佳; 王栋民; 李景中. 拉萨市棘球蚴病流行现状分析. 中国寄生虫学与寄生虫病杂志 2018.(La-Mu, C.; Yan, X.L.; Wang-Jiu, D.; Long, Z.Y.; Qu-Zhen, D.; Jia-Jia, A.I.; Wang, D.M.; Jing-Zhong, L.I. Epidemiological status of echinococcosis in lhasa city. Chinese Journal of Parasitology & Parasitic Diseases 2018.)
34. 贡桑曲珍; 李斌; 陈伟奇; 嘎松; 索郎旺杰; 王栋民; 康珠益西; 李景中. 昌都市棘球蚴病流行现状分析. 中国寄生虫学与寄生虫病杂志 2018.(Qu-Zhen, G.; Bin, L.I.; Chen, W.Q.; Song, G.A.; Wang-Jie, S.; Wang, D.M.; Yi-Xi, K.; Jing-Zhong, L.I. Prevalence of echinococcosis in changdu city. Chinese Journal of Parasitology & Parasitic Diseases 2018.)
